# Differences in the Nonuse of any Contraception and Use of Specific Contraceptive Methods in HIV Positive and HIV Negative Rwandan Women

**DOI:** 10.1155/2012/367604

**Published:** 2012-12-17

**Authors:** Adebola A. Adedimeji, Donald R. Hoover, Qiuhu Shi, Mardge H. Cohen, Tracy Gard, Kathryn Anastos

**Affiliations:** ^1^Centre for Public Health Sciences, Albert Einstein College of Medicine, Mazer 515, 1300 Morris Park Avenue, Bronx, NY 10461, USA; ^2^Department of Epidemiology and Population Health, Albert Einstein College of Medicine, Bronx, NY 10461, USA; ^3^Department of Statistics and Institute for Health, Health Care Policy and Aging Research, Rutgers University, New Brusnwick, NJ 08901, USA; ^4^New York Medical College, Valhalla, NY 10595, USA; ^5^Department of Medicine, Stroger (Cook County) Hospital and Rush University, Chicago, IL 60612, USA; ^6^Department of Psychiatry and Behavioral Sciences, Albert Einstein College of Medicine, Bronx, NY 10461, USA; ^7^Department of Medicine, Montefiore Medical Centre, Bronx, NY 10467, USA

## Abstract

Contraception can reduce the dual burden of high fertility and high HIV prevalence in sub-Sahara Africa, but significant barriers remain regarding access and use. We describe factors associated with nonuse of contraception and with use of specific contraceptive methods in HIV positive and HIV negative Rwandan women. Data from 395 HIV-positive and 76 HIV-negative women who desired no pregnancy in the previous 6 months were analyzed using univariate and multivariate logistic regression models to identify clinical and demographic characteristics that predict contraceptive use. Differences in contraceptive methods used were dependent on marital/partner status, partner's knowledge of a woman's HIV status, and age. Overall, condoms, abstinence, and hormonal methods were the most used, though differences existed by HIV status. Less than 10% of women both HIV+ and HIV− used no contraception. Important differences exist between HIV-positive and HIV-negative women with regard to contraceptive method use that should be addressed by interventions seeking to improve contraceptive prevalence.

## 1. Background

Sub-Sahara (SSA) Africa has the highest population growth rate and the greatest burden of HIV infection in the world. Barrier and hormonal contraceptive methods offer feasible means to address the dual burden of high fertility and high HIV prevalence in the region. While contraceptive use among SSA women has increased in the past decade, disparities remain between and within countries and use still remains below 20% in many countries [1]. Barriers to higher usage include poor access, cost, inadequate health infrastructure, and sociocultural values supporting high fertility [1–3]. 

Rwanda, the most densely populated country in Africa has an HIV prevalence rate of 2.9% [4], mostly among childbearing age women [5]. Expanded access to free antiretroviral therapy contributed to significant gains in health and quality of life of people living with HIV/AIDS (PLHA). These gains in health, coupled with a low risk of mother-to-child transmission to less than 1% [6] and the high value on fertility have altered the context of fertility decision making for many PLHAs. This context is mediated by individual, interpersonal, medical, structural, and cultural factors [7–12] thus making fertility control a major policy and service delivery issue in the care of PLHA [13]. 

Rwandan women face significant obstacles to access and use of contraception. Ayad and Hong [14] reported that while contraceptive prevalence rate among Rwandan women increased substantially between 2005 and 2008 from 13% to 36%, the level of unmet need also increased within the same period. Other studies also reported low use and high levels of unmet need [3, 15–17] despite a prevalent desire to limit fertility [18–20]. 

Unintended pregnancy accounts for 15–58% of births in countries with high HIV burden [21] including Rwanda [22]. Unintended pregnancies, which may directly result from unmet needs underscore the importance of understanding contraceptive decision-making among HIV positive (HIV+) women who may be more interested in preventing pregnancy than in preserving their own health and eliminating the risk of transmitting the virus to their sex partners [15, 23–25]. Therefore access to and use of safe, effective contraception, as advocated in the Glion Call to Action [5] is critical for this population. Indeed, the effectiveness of voluntary contraception among HIV+ women has been well documented in literature [21, 26–28] with some estimates suggesting that current contraceptive use among HIV+ women may already be preventing as many as 220,000 HIV+ births annually in high prevalent countries. 

There is a low but increasing rate of modern contraceptive use among Rwandan women, however little is known about the predictors of contraceptive practice. To aid policy and program planning in meeting the reproductive needs and rights of HIV+ Rwandan women [15], it is important to identify and understand factors that influence contraceptive choices and use and how this is similar to or different from those of HIV negative (HIV−) women. This paper explores and describes the factors associated with nonuse of any contraception and use of specific contraceptive methods between HIV+ and HIV− Rwandan women. We hypothesized that HIV status would significantly determine the type of contraceptive methods favored by Rwandan women, for instance that HIV+ women would prefer barrier methods such as condoms while HIV− women will prefer hormonal methods. 

## 2. Methods

The Rwanda Women's Interassociation Study and Assessment (RWISA) is a prospective observational cohort study of HIV infected and uninfected Rwandan women. Details of the study methods (including participants, recruitment methods, eligibility criteria, and informed consent process) have been previously described [29, 30]. In 2005, 710 HIV+ and 226 HIV− women enrolled in RWISA, recruited through grassroots women's associations and HIV care sites in Kigali. Eligibility criteria included living in Rwanda and aged >15 years during the 1994 genocide, agreeing to be tested for HIV and willingness to travel to the study site to participate in follow-up visits. The Rwandan National Ethics Committee and the Montefiore Medical Center Institutional Review Board approved the study protocol and procedures.

By design, 50% of both HIV+ and HIV− participants reported rape during the 1994 genocide. During the enrollment visit, participants provided historical information, underwent physical and gynecological examination, and provided blood, urine and gynecological specimens. Interviews were conducted in Kinyarwanda by trained interviewers with nursing or trauma counseling backgrounds. The population for this consisted of 395 HIV+ and 76 HIV− women who reported at enrollment that they desired not to become pregnant. 

### 2.1. Measures

Participants provided demographic, medical, psychosocial, and behavioral information regarding clinical status, disease progression, HIV-1 exposure risks, quality of life, symptoms of depression and posttraumatic stress disorder (PTSD), contraceptive practice, and trauma experience during the 1994 genocide. 

The following variables were created for this analysis: HIV/CD4 Status (HIV−, HIV+ and CD4 < 200, HIV+ and CD4 200–350, and HIV+ and CD4 > 350); partner knowledge of HIV status (HIV+ participant whose partner knows her status, HIV+ participant whose partner does not know, HIV− participant whose partner knows, and HIV− participant whose partner does not know). Women were asked if they had used each of the following contraceptive methods at least once in the previous six months; oral contraceptives, implantable or depot/injected progesterone, intrauterine device, diaphragm or cervical cap, vaginal creams/jellies/foams, or the sponge, rhythm or withdrawal, emergency contraception, male or female condoms, and abstinence. The following methods were combined together to form “hormonal contraception”: oral contraceptives, implantable, or depot progesterone. Respondents were not required to report frequency of contraceptive usage. 

### 2.2. Statistical Analysis

We compared HIV+ and HIV− women by categorized characteristics using exact tests for statistical significance. Univariate and multivariate logistic regression models were fit to determine associations with the probability of (i) not practicing at least one contraceptive method and (ii) practicing contraceptive methods in the following categories: abstinence, hormonal, and condoms. Multivariate models were fit using stepwise selection among all variables in Table 2 with a *P* value for entry of 0.05 and a *P* value for removal of >0.1. 

## 3. Results

Table 1 shows demographic and clinical characteristics for 471 participants. Among HIV+ women, nearly one-third had a CD4 cell count of less than 200 cells/*μ*L. Forty-seven percent of all respondents were married or currently living with a partner. The majority was of low socioeconomic status; 37% of HIV− women and 27% of HIV+ women were employed and on a monthly income of less than 10,000 Rwandan Franc (~US$17.40). Sixty percent of HIV− and 58% of HIV+ women reported that partners were aware of their HIV sero-status.

Table 2 presents univariate and multivariate analyses of demographic and clinical characteristics associated with the use of abstinence as a contraceptive method in the prior 6 months. Abstinence was a commonly used method, reported by about 40% of HIV+ and HIV− women. Use of abstinence was significantly associated with partner's knowledge of respondent's HIV status, being married or living with a partner, income and ever having had sex for cash. HIV+ women whose partners were not aware of their status were more likely (OR = 3.83) than HIV+ women whose partners were aware of their status to use abstinence as a method of contraception; women who were married or living with a partner were significantly less likely to report using abstinence (OR = 0.06) than those who were not married or living with a partner. Women who had ever exchanged sex for cash were also more likely than those who had not to report abstinence as a means of contraception (OR = 1.61).

In the final stepwise multivariate model, marital status, HIV status/partner's knowledge of status, and ever exchanging sex for money were independently associated with abstinence. Women who were married or living with a partner were 20-fold less likely to report using abstinence: adjusted odds ratio (aOR = 0.05). Compared to HIV+ women whose partners knew their HIV status, HIV+ women whose partners *did not* know their status (aOR = 1.89) and HIV− women whose partners *did* know their status (aOR = 2.83) were more likely to use abstinence. Ever exchanging sex for money which was associated with *more* abstinence in unadjusted analysis was now significantly associated with *less* abstinence in the multivariate model. This change in direction of the association occurred because one variable in the model, married/living with a partner, was strongly negatively associated with both abstinence and having exchanged sex for money.

Overall, condom use (both male and female) was the most frequently reported method of contraception. While condom use differed by HIV status (58% of HIV+ versus 18% of HIV− OR = 6.06) due to differences in male condom usage, female condom use reported by only 8 women did not differ by HIV status, although with the small number of users there was no power to detect any difference. Univariate analysis in Table 3 shows significant associations between condom use in the previous six months and HIV status, number of living children, partner's knowledge of HIV status, age, and income. For example, HIV+ women were 3-fold more likely than HIV− women to report condom use; women with higher incomes were significantly more likely (OR = 2.00) to report condom use than were women with lower incomes. HIV+ women whose partners knew their sero-status were more likely to report condom use than HIV+ women whose partners did not know their status and HIV− women regardless of partners' knowledge of their status. Women who had no children were more likely to report condom use than were those with at least one child (*P* < 0.001 for all previous associations). 

Clinical and demographic characteristics that were significantly associated independently with *condom use* in adjusted models included marital status (aOR = 3.13 for those married or living with a partner compared to those not) and age (aOR = 3.35 for those less than 30 and aOR = 2.15 for those between 30 and 40 years, resp., compared to women >40 years). Similarly, current employment (aOR = 1.88 compared to women not employed) and history of trading sex for cash (aOR = 2.53 compared to no history) were independently associated with increased likelihood of condom use. Compared to HIV+ women whose partners knew their status, all other groups were independently less likely to report condom use for contraceptive purposes: (aOR = 0.35 for HIV+ women whose partners did not know their HIV status; aOR = 0.16 for HIV− women whose partners knew their status and aOR = 0.03 for HIV− women whose partners did not know their status), respectively.

About 10% of HIV+ and 20% of HIV− women reported use of hormonal methods. In Table 4, univariate analysis shows that use of hormonal contraception was significantly associated with a woman's HIV/CD4 status category, her HIV status/partner's knowledge of that status, being married or living with a partner, and having an income of at least 35,000 FRW. Women who were HIV+ and whose CD4 was greater than 200 cells/*μ*L were less likely than HIV− women to report using hormonal contraceptive methods (OR = 0.34 and 0.35 for women with 200–350 and >350 cells/*μ*L, resp.). In multivariate analysis, hormonal contraceptive use was independently more common in women who were married or living with a partner (aOR = 2.67) and in HIV− women whose partners knew their status (aOR = 4.13 compared to HIV+ women whose partners know their status, resp.). 

Table 5 shows few women (5.6% of HIV− and 7.2% of HIV+) reported using no contraceptive method during the previous 6 months. Being married or living with a partner was significantly associated with *no contraceptive use* in the past 6 months (OR = 2.25) compared to women not married or living with a partner. Age and marital status had significant independent associations with *no contraceptive use* in the multivariate analysis. Women reporting *no contraceptive use* were independently more likely to be married or living with a partner (aOR = 2.55) compared with those who were not. Women younger than 30 years were more likely to report *no contraceptive use* compared to women 30–40 (aOR = 0.27) and >40 (aOR = 0.37), respectively. It should be noted that 11 women (4 HIV+, 7 HIV−) had a surgical method (hysterectomy, tubal ligation, and ovary removal). While this usage was higher for HIV− women (*P* = 0.0003), the numbers were too small to allow further analysis.

A little over 10% of participants reported using multiple methods but these did not statistically differ by HIV status; 9.2% of HIV− compared with 14.7% of HIV+ women (*P* = 0.21 exact test). Condoms were almost always one of the multiple methods reported; 47 women reported using condoms and abstinence while 21 women reported using condoms and hormonal methods. This perhaps reflects that abstinence was used if condoms were not available and that condoms were most likely used as a backup to hormonal methods as well as to prevent STD transmission. 

## 4. Discussion 

In this study of Rwandan women desiring not to become pregnant we found that over 90% of both HIV infected and uninfected women described using some form of contraception, with condoms being the most prevalent method used. We also found that HIV status combined with partner knowledge of status was more strongly associated with the type of contraceptive method used than HIV status alone or CD4 count. Specifically, HIV+ women whose partners knew their status were two to ten times more likely to report condom use when compared to other groups. This suggests that disclosure of a positive HIV status to a partner may be an important contributor to pregnancy prevention through condom use in addition to partner protection from HIV. Marital status and age were also significantly associated with contraceptive method used. 

We also found that older women were less likely to use condoms, suggesting perhaps a need to educate older women on condom use to prevent HIV transmission as well as pregnancy, in addition to or instead of using other methods. Apart from providing effective contraception, condoms also reduce the risk of HIV transmission and can be used concurrently with other contraceptive methods. The effectiveness of condoms, however, depends on correct and consistent use as well as acceptance by male partners. Previous studies have highlighted several socioeconomic, cultural and behavioral factors that inhibit condom use. For instance, in the case of male condoms, many men in SSA may interpret a request to use condoms as an insult, a sign of mistrust, and a hindrance to sexual fulfillment [31–35]. Additional determinants of condom use are female decision-making power [36, 37], socioeconomic factors, access to and availability of condoms [38, 39] technical issues with substandard condoms [40–43], and myths and misconceptions about condoms and fertility aspirations. It is important therefore that efforts to increase condom use address the barriers that have been highlighted in the literature.

Studies of HIV discordant couples have shown a marked increase in the proportion using condoms, following behavioral interventions, to prevent transmitting the virus between partners [3, 44–46], and not to prevent pregnancy. These studies also show that condom use was more consistent when the man was HIV− compared to when the woman was HIV−. In the current study, the strong association observed between condom use and HIV status, number of living children, partners' knowledge of HIV status, and socioeconomic status also suggests that disease prevention not pregnancy prevention may be the primary reason for condom use. For example, condom use was significantly higher among HIV+ women whereas it was lower among women who already had one or more children. Women with a history of sex in exchange for cash were also more likely to use condoms, which may indicate a desire to prevent disease and perhaps pregnancy to limit the number of dependents. Furthermore, that income status was associated with condom use could point to issues of access and affordability among those with low or no income.

Hormonal contraceptive methods are among the most effective contraception available to women who desire to control when and how to have children. Despite their effectiveness, only 21% of HIV− women and 10% of HIV+ women reported using hormonal methods. Some studies [47] have suggested that among Rwandan women, low use of hormonal contraceptive methods may be due to lack of access and availability, low knowledge, and cost. In the case of HIV+ women, studies [48–52] suggest that the low use of hormonal methods may be due to misconceptions leading to inconsistent use, concerns regarding hormonal methods' possible contribution to HIV transmission to sex partners or accelerated HIV disease progression and possible interaction with antiretroviral agents. The concerns on the interaction between hormonal methods with HIV acquisition and HIV-associated disease progression are areas of active research [53, 54]. Prior literature has been contradictory with some studies finding higher rates of HIV-acquisition [55, 56] and more rapid disease progression [57, 58] in users of hormonal contraception, and some finding no effect [59, 60]. However, since the efficacy of estrogen-containing contraception can be compromised by many of the antiretrovirals [59], it is understandable that HIV+ women are less likely to use hormonal methods. 

Respondents were not asked about the frequency of contraceptive use, especially of self-reported condom use and abstinence, therefore this is one limitation of the study. It is also not possible to separate use of abstinence and condoms to prevent pregnancy from the use of these methods to prevent STD, although all of the study participants stated that they did not want to become pregnant. While generalizability of this population to other women is not certain, this group represents an important group of HIV infected and uninfected women.

Of note, about 10% of the HIV+ and HIV− women in this analysis did not use any method of contraception despite expressing a desire to not get pregnant. Younger age and being married or living with a partner were predictors of nonuse of any contraceptive method in univariate and/or multivariate models. Although there is evidence, which suggests that an increasing number of Rwandan women now have access to and use modern contraceptive methods [1, 61] a considerable proportion of women may still experience barriers to access and use. Further studies should explore the characteristics and reasons why women in this population are not using contraception.

## 5. Conclusion

There is abundant literature [62–70] describing the pathways through which the HIV epidemic has contributed to population declines or indeed, a demographic transition in SSA, resulting in a fall in demand for children, significantly lowered fertility desires and increased contraceptive use. The Rwanda Demographic and Health Surveys [61] show that the proportion of women reporting contraceptive use rose from 17% in 2005 to 36% in 2008. About 90% of women in this study reported using contraception although the population is by no means representative since they were recruited from grass roots organizations and HIV clinics. 

Condoms and abstinence were the most commonly used methods of contraception among the women in this study. Women who were married or living with their partners were far less likely to abstain and far more likely to use condoms. Older women were far less likely to use condoms. Women who were HIV+ and whose partners were aware of this were far more likely to use condoms. Despite their effectiveness, use of hormonal methods is still low, highlighting issues of availability, access or cost and possibly concerns about the side effects of hormonal methods, especially among HIV+ women. Moreover, the different contraceptive needs of HIV+ and HIV− women including preventing transmission of HIV and sexually transmitted diseases should be recognized when planning interventions to improve contraceptive use. 

## Figures and Tables

**Table 1:** Demographic and clinical characteristics of 471 women who reported wanting to prevent pregnancy.

Characteristics	HIV-negative *n* = 76	HIV-positive *n* = 395	P value
Age			
<30	17 (22.4%)	97 (24.6%)	0.001
30–40	34 (44.8%)	245 (62.0%)	
>40	25 (32.9%)	53 (13.4%)	
Married and living with partner			
Yes	39 (51.3%)	183 (46.3%)	0.425
No	37 (38.7%)	212 (53.7%)	
Currently pregnant			
Yes	1 (1.3%)	2 (0.5%)	0.419
No	75 (98.7%)	391 (99.5%)	
Employment status			
Yes	27 (37.0%)	103 (26.6%)	0.071
No	46 (63.0%)	284 (73.4%)	
Monthly income			
<10,000	25 (34.2%)	121 (28.1%)	0.033
10–35,000	28 (38.4%)	208 (52.8%)	
>35,000	20 (27.4%)	65 (16.5%)	
Experienced genocidal rape			
Yes	46 (61.3%)	193 (49.3%)	0.055
No	29 (38.7%)	199 (50.7%)	
Number of living children			
0	13 (17.2%)	111 (28.1%)	0.001
1-2	16 (21.0%)	136 (34.4%)	
3-4	31 (40.8%)	108 (27.4%)	
5+	16 (21.0%)	40 (10.1%)	
HIV/CD4 Status			
HIV−	76 (100.0%)	0	0.001
HIV+ CD4 < 200	0	106 (26.8%)	
HIV+ CD4 200–350	0	152 (38.5%)	
HIV+ CD4 > 350	0	137 (34.7%)	
Partner knowledge of HIV status			
HIV+ Partner knows	0	222 (58.4%)	0.001
HIV+ Partner does not know	0	158 (41.6%)	
HIV− Partner knows	43 (60.6%)	0	
HIV− Partner does not know	28 (39.4%)	0	
Ever had sex for cash			
Yes	14 (18.4%)	94 (23.9%)	0.302
No	62 (81.6%)	300 (76.1%)	

**Table 2:** Univariate and multivariate analysis of demographic and clinical characteristics associated with abstinence.

Variable	Abstinence		
	Proportion practicing method *n* (%)	Univariate OR (95% CI)	Multivariate Adjusted OR (95% CI)^a^
HIV Status			
HIV−	32 (42.1%)	(*r*)	
HIV+	152 (38.7%)	0.87 (0.53–1.43)	
HIV/CD4 Status			
HIV−	32 (42.1%)	(*r*)	
HIV+ CD4 < 200	31 (44.8%)	0.57 (0.31–1.06)	
HIV+ CD4 on [200, 500]	60 (39.7%)	0.91 (0.52–1.59)	
HIV+ CD4 ≥ 350	61 (29.2%)	1.12 (0.63–1.97)	
Number of living children			
0	55 (44.7%)	(*r*)	
1-2	63 (41.7%)	0.89 (0.55–1.43)	
3-4	48 (34.5%)	0.65 (0.40–1.07)	
5+	18 (32.1%)	0.59 (0.30–1.14)	
HIV Status/Partner knowledge			
HIV+ Partner knows	50 (22.6%)	(*r*)	(*r*)
HIV+ Partner does not know	92 (58.6%)	3.83 (2.50–5.87)***	1.89 (1.13–3.16)*
HIV− Partner knows	19 (44.2%)	2.14 (1.10–4.17)*	2.83 (1.22–6.56)*
HIV− Partner does not know	8 (28.6%)	1.08 (0.45–2.58)	0.68 (0.25–1.87)
Age			
>30	40 (35.1%)	0.57 (0.32–1.02)	
30–40	106 (38.3%)	0.65 (0.39–1.08)	
40+	38 (48.7%)	(*r*)	
Married or living with partner			
No	162 (65.3%)	(*r*)	(*r*)
Yes	22 (9.9%)	0.06 (0.04–0.10)***	0.05 (0.03–0.09)
Genocidal rape			
No	89 (39.2%)	(*r*)	
Yes	35 (39.9%)	1.03 (0.71–1.49)	
Employed			
No	128 (39.0%)	(*r*)	
Yes	60 (38.5%)	0.98 (0.64–1.48)	
Income			
0–10,000	79 (54.5%)	(*r*)	
10,001–35,000	85 (36.1%)	0.46 (0.30–0.70)***	
35,001+	17 (20.0%)	0.20 (0.11–0.38)***	
Ever had sex for cash			
No	120 (35.9%)	(*r*)	(*r*)
Yes	63 (47.4%)	1.61 (1.07–2.41)*	0.59 (0.35–0.98)*

^a^Models built by stepwise selection among all variables in this table with a *P* value for entry of 0.05 and a *P* value for removal of >0.1.

*P < 0.05, **P < 0.01, ***P < 0.001.

**Table 3:** Univariate and multivariate analysis of demographic and clinical characteristics associated with condom use.

Variable	Condom		
	Proportion practicing method *n* (%)	Univariate OR (95% CI)	Multivariate Adjusted OR (95% CI)^a^
HIV Status			
HIV−	14 (18.4%)	(*r*)	
HIV+	227 (57.8%)	6.06 (3.28–11.18)***	
HIV/CD4 Status			
HIV−			
HIV+ CD4 < 200	84 (46.1%)	(*r*)	
HIV+ CD4 on [200, 500]	93 (61.6%)	1.87 (1.21–2.90)**	1.19 (0.67–2.14)
HIV+ CD4 ≥ 350	64 (47.1%)	**1.04 (0.66–1.62)**	0.62 (0.35–1.11)
Number of living children			
0	78 (62.9%)	(*r*)	
1-2	74 (49.0%)	0.57 (0.35–0.92)	
3-4	62 (44.9%)	0.48 (0.29–0.79)*	
5+	27 (48.2%)	0.55 (0.29–1.04)**	
HIV Status/Partner knowledge			
HIV+ Partner knows	160 (72.4%)	(*r*)	(*r*)
HIV+ Partner does not know	60 (38.2%)	0.27 (0.18–0.41)***	0.34 (0.21–0.55)***
HIV− Partner knows	11 (25.6%)	0.15 (0.07–0.31)***	0.15 (0.06–0.35)***
HIV− Partner does not know	2 (7.1%)	0.03 (0.01–0.14)***	0.03 (0.01–0.13)***
Age			
>30	67 (58.8%)	3.63 (1.96–6.73)***	3.59 (1.75–7.34)
30–40	152 (54.9%)	3.10 (1.79–5.35)***	2.24 (1.20–4.20)
40+	22 (28.2%)	(*r*)	(*r*)
Married or living with partner			
No	101 (40.7%)	(*r*)	(*r*)
Yes	140 (63.3%)	2.52 (1.73–3.65)	3.05 (1.86–5.00)***
Genocidal rape			
No	114 (50.0%)	(*r*)	
Yes	125 (52.7%)	1.12 (0.78–1.61)	
Employed			
No	165 (50.1%)	(*r*)	(*r*)
Yes	74 (57.4%)	1.34 (0.89–2.02)	1.86 (3.05)*
Income			
0–10,000	60 (41.4%)	(*r*)	
10,001–35,000	135 (57.4%)	2.00 (1.32–3.04)**	
35,001+	46 (54.1%)	1.75 (1.02–3.00)*	
Ever had sex for cash			
No	160 (47.9%)	(*r*)	(*r*)
Yes	80 (60.1%)	1.64 (1.09–2.47)	2.54 (1.51–4.26)

^a^Models built by stepwise selection among all variables in this table with a *P* value for entry of 0.05 and a *P* value for removal of >0.1.

*P < 0.05, **P < 0.01, ***P < 0.001.

**Table 4:** Univariate and multivariate analysis of demographic and clinical characteristics associated with hormonal contraceptive use.

Variable	Hormonal		
	Proportion practicing method *n* (%)	Univariate OR (95% CI)	Multivariate adjusted OR (95% CI)^a^
HIV Status			
HIV−	16 (21.6%)	(*r*)	
HIV+	40 (10.2%)	0.41 (0.22–0.79)**	
HIV/CD4 Status			
HIV−	16 (21.6%)	(*r*)	
HIV+ CD4 < 200	15 (14.1%)	0.60 (0.27–1.30)	
HIV+ CD4 on [200, 500]	13 (8.7%)	0.34 (0.16–0.76)**	
HIV+ CD4 ≥ 350	12 (8.9%)	0.35 (0.16–0.80)*	
Number of living children			
0	10 (8.1%)	(*r*)	
1-2	19 (5.7%)	1.63 (0.73–3.64)	
3-4	7 (16.7%)	1.97 (0.88–4.38)	
5+	7 (12.5%)	1.61 (0.58–4.49)	
HIV Status/Partner knowledge			
HIV+ Partner knows	30 (13.6%)	(*r*)	(*r*)
HIV+ Partner does not know	9 (5.8%)	0.41 (0.19–0.89)*	0.60 (0.27–1.34)
HIV− Partner knows	7 (16.7%)	1.35 (0.55–3.30)	1.40 (0.57–3.48)
HIV− Partner does not know	9 (33.3%)	3.37 (1.39–8.17)**	4.13 (1.65–10.37)**
Age			
>30	15 (13.2%)	1.31 (0.53–3.25)	
30–40	33 (12.0%)	1.18 (0.52–2.67)	
40+	8 (10.4%)	(*r*)	
Married or living with partner			
No	17 (6.9%)	(*r*)	(*r*)
Yes	39 (17.9%)	2.95 (1.61–5.38)***	2.67 (1.40–5.08)**
Genocidal rape			
No	27 (11.9%)	(*r*)	
Yes	27 (11.5%)	0.96 (0.54–1.69)	
Employed			
No	36 (11.0%)	(*r*)	
Yes	19 (14.5%)	1.40 (0.77–2.55)	
Income			
0–10,000	12 (8.3%)	(*r*)	
10,001–35,000	25 (10.8%)	1.26 (0.62–2.56)	
35,001+	18 (21.4%)	2.85 (1.32–6.17)**	
Ever had sex for cash			
No	41 (12.4%)	(*r*)	
Yes	15 (11.4%)	0.91 (0.48–1.70)	

^a^Models built by stepwise selection among all variables in this table with a *P* value for entry of 0.05 and a *P* value for removal of >0.1.

*P < 0.05, **P < 0.01, ***P < 0.001.

**Table 5:** Univariate and multivariate analysis of demographic and clinical characteristics of nonuse of contraception^1^.

Variable	No contraception		
	Proportion practicing method *n* (%)	Univariate OR (95% CI)	Multivariate adjusted OR (95% CI)^a^
HIV Status			
HIV−	4 (5.6%)	(*r*)	
HIV+	28 (7.2%)	1.30 (0.44–3.81)	
HIV/CD4 Status			
HIV−	4 (5.6%)	(*r*)	(*r*)^b^
HIV+ CD4 < 200	9 (8.5%)	1.55 (0.46–5.25)	2.06 (0.58–7.34)
HIV+ CD4 on [200, 500]	4 (2.7%)	0.46 (0.11–1.89)	0.57 (0.13–2.40)
HIV+ CD4 ≥ 350	15 (11.2%)	2.11 (0.57–6.62)	3.13 (0.94–10.43)
Number of living children			
0	6 (4.9%)	(*r*)	
1-2	9 (6.1%)	1.26 (0.44–3.65)	
3-4	12 (8.8%)	1.87 (0.68–5.15)	
5+	5 (8.9%)	1.90 (0.55–6.50)	
HIV Status/Partner knowledge			
HIV+ Partner knows	15 (6.8%)	(*r*)	
HIV+ Partner does not know	13 (8.4%)	1.39 (0.64–3.01)	
HIV− Partner knows	1 (2.3%)	0.36 (0.05–2.79)	
HIV− Partner does not know	3 (13.0%)	2.26 (0.60–8.47)	
Age			
>30	5 (4.5%)	0.35 (0.11–1.08)	0.27 (0.08–0.87)*
30–40	18 (6.6%)	0.53 (0.23–1.22)	0.37 (0.15–0.92)*
40+	9 (11.8%)	(*r*)	(*r*)
Married or living with partner			
No	14 (6.2%)	(*r*)	(*r*)
Yes	17 (7.3%)	2.25 (1.06–4.78)*	2.55 (1.17–5.58)*
Genocidal rape			
No	14 (6.2%)	(*r*)	
Yes	17 (7.3%)	1.18 (0.57–2.46)	
Employed			
No	23 (7.1%)	(*r*)	
Yes	7 (5.5%)	0.75 (0.31–1.80)	
Income			
0–10,000	11 (7.7%)	(*r*)	
10,001–35,000	14 (6.0%)	0.80 (0.35–1.81)	
35,001+	7 (8.4%)	1.14 (0.42–3.06)	
Ever had sex for cash			
No	27 (8.3%)	(*r*)	
Yes	5 (3.8%)	0.44 (0.16–1.16)	

^a^Models built by stepwise selection among all variables in this table with a *P* value for entry of 0.05 and a *P* value for removal of >0.1.

^b^Even though all 95% CIs contain 1, the overall *P* value for significance of HIV CD4 status considering all categories simultaneously is <0.1 by Wald test.

*P < 0.05, **P < 0.01, ***P < 0.001.

^1^Thus category excludes women who use surgical methods in addition to the other methods in the previous tables; condoms, hormones, and abstinence.
